# Large language models and GenAI in education: Insights from Nigerian in-service teachers through a hybrid ANN-PLS-SEM approach

**DOI:** 10.12688/f1000research.161637.1

**Published:** 2025-03-04

**Authors:** Musa Adekunle Ayanwale, Owolabi Paul Adelana, Nurudeen Babatunde Bamiro, Stella Oluwakemi Olatunbosun, Kabir Oluwatobi Idowu, Kayode A. Adewale

**Affiliations:** 1 Department of Mathematics, Science and Technology Education, Faculty of Education, University of Johannesburg, Auckland Park, South Africa; 2 Institute of Educational Technology, The Open University, Milton Keynes, UK; 3 Department of Economics, Faculty of Management and Economics, Universiti Pendidikan Sultan Idris, Tanjong Malim, Malaysia; 4 Department of Educational Psychology, Faculty of Education, University of Johannesburg, Auckland Park, South Africa; 5 Department of Mathematics, Faculty of Science, Purdue University, West Lafayette, USA; 6 Department of Counseling Psychology and Educational Foundations, College of Specialised and Professional Education, Tai Solarin University of Education, Ijebu Ode, Nigeria

**Keywords:** Generative AI in teaching, large language models, PLS-SEM, artificial neural networks, in-service teacher training

## Abstract

**Background:**

The rapid integration of Artificial Intelligence (AI) in education offers transformative opportunities to enhance teaching and learning. Among these innovations, Large Language Models (LLMs) like ChatGPT hold immense potential for instructional design, personalized learning, and administrative efficiency. However, integrating these tools into resource-constrained settings such as Nigeria presents significant challenges, including inadequate infrastructure, digital inequities, and teacher readiness. Despite the growing research on AI adoption, limited studies focus on developing regions, leaving a critical gap in understanding how educators perceive and adopt these technologies.

**Methods:**

We adopted a hybrid approach, combining Partial Least Squares Structural Equation Modelling (PLS-SEM) and Artificial Neural Networks (ANN) to uncover both linear and nonlinear dynamics influencing behavioral intention (BI) of 260 Nigerian in-service teachers regarding ChatGPT after participating in structured training. Key predictors examined include Perceived Ease of Use (PEU), Perceived Usefulness (PUC), Attitude Towards ChatGPT (ATC), Your Colleagues and Your Use of ChatGPT (YCC), Technology Anxiety (TA), Teachers’ Trust in ChatGPT (TTC), and Privacy Issues (PIU).

**Results:**

Our PLS-SEM results highlight PUC, TA, YCC, and PEU, in that order of importance, as significant predictors, explaining 15.8% of the variance in BI. Complementing these, ANN analysis identified PEU, ATC, and PUC as the most critical factors, demonstrating substantial predictive accuracy with an RMSE of 0.87. This suggests that while PUC drives adoption, PEU and positive attitudes are foundational in fostering teacher engagement with AI technologies.

**Conclusion:**

Our results highlight the need for targeted professional development initiatives to enhance teachers’ digital competencies, reduce technology-related anxiety, and build trust in AI tools like ChatGPT. Our study offers actionable insights for policymakers and educational stakeholders, emphasizing the importance of fostering an inclusive and ethical AI ecosystem. We aim to empower teachers and support AI-driven educational transformation in resource-limited environments by addressing contextual barriers.

## 1. Introduction

Large Language Models (LLMs) are advanced Artificial Intelligence (AI) technologies that are trained on extensive datasets to process and generate natural language responses to text inputs. LLMs rely on key components such as transformers for input representation, deep and self-supervised learning, pre-training, and fine-tuning ( Zhou et al., 2024; Guo et al., 2022). LLMs have found their ways into almost all human endeavours, including education. As Large Language Models (LLMs), for instance, Generative Artificial Intelligence (GenAI) deeply find their ways into education, their transformative potentials in education offer unprecedented opportunities via automated assessment, personalized learning, and enhanced educational resources to help educators improve their practices in a wide range of pedagogical scenarios ( Pozdniakov et a., 2024). As artificial intelligence (AI), especially GenAI continues to emerge and reshape education by enhancing content creation ( Denny et al., 2023), ease of creating multiple-choice questions ( Bulathwela et al., 2023), AI-generated educational video clips ( Leiker et al., 2023), and adaptive learning tools ( Jury et al., 2024). GenAI also supports assessments through feedback automation for students ( Han et al., 2023) while also improving personalized and innovative educational experiences for both students and their teachers.

Empirical reports support GenAI and related AI-based technologies capacity to revolutionize traditional pedagogical approaches thereby enabling more adaptive and responsive teaching methods at all levels of education ( Biswas, 2023; Dowling & Lucey, 2023; El Naqa et al., 2023; Huang et al., 2023; Lund et al., 2023; Meskó & Topol, 2023; Baclic et al., 2020). However, the successful integration of these technologies in educational domains is dependent upon teachers’ acceptance because their perspectives and behavioural intentions to use the technologies play critical roles concerning their innovative adoption ( Ayanwale et al., 2022).

Understanding teachers’ intention to integrate and use GenAI in education is important for facilitating effective implementation ( Aghaziarati et al., 2023; Ayanwale et al., 2022) especially in resource-constrained environments such as Nigeria where the application and use of GenAI by teachers is still limited due to systemic challenges. Teachers in these settings often face inadequate access to digital infrastructure, insufficient training, ongoing digital inequalities, data privacy, technological infrastructure, and the importance of aligning AI tools with pedagogical goals ( Nguyen et al., 2023b; Shahzad et al., 2024; Walter, 2024) all of which hinder the effective use of GenAI ( Essien et al., 2024). As education undergoes digital transformation leading to innovative learning spaces in which humans and machines collaborate based on AI’s transformative infiltration into education, the need arises to further examine teachers’ intention to use the technology going forward ( Lu et al., 2024). In view of this, we hypothesize that Perceived Ease of Use (PEU), Perceived Usefulness (PUC), Attitude Toward ChatGPT (ATC), Teachers’ Trust in ChatGPT (TTC), and Your Colleagues and Your Use of ChatGPT (YCC) positively influence teachers’ Behavioral Intention (BI) to adopt ChatGPT. Conversely, we propose that Technology Anxiety (TA) and Privacy Issues (PIU) negatively impact BI.

Our study is anchored on hypotheses that guide the examination of both linear and nonlinear relationships among variables. Following structured training on ChatGPT applications, we assess Nigerian in-service teachers’ perspectives using a hybrid methodology. By integrating Partial Least Squares Structural Equation Modeling (PLS-SEM) and Artificial Neural Networks (ANN), we identify key drivers and obstacles to ChatGPT adoption. PLS-SEM facilitates the analysis of linear relationships, reliability assessment, and evaluation of direct and indirect effects, while ANN captures intricate nonlinear interactions and prioritizes predictor importance. This combined approach offers a comprehensive understanding of factors influencing teachers’ readiness and intention to incorporate AI tools into their educational practices. Our study there examines in-service teachers’ behavioural intention to use LMM’s GenAI from which we construct a model of teachers’ intention to use the technology in education by drawing on the TAM framework to explore the influencing factors of their intention. In view of this, our study addresses the contextual challenges of AI adoption in resource-limited settings and provides actionable insights to empower teachers, promoting equitable, innovative, and sustainable educational practices in developing countries.

## 2. Review of related literature and hypothesis development

### 2.1 Large language models

The rapid development of artificial intelligence (AI) technologies in recent years, especially the widespread use of large language models (LLMs), has sparked much scholarly discussion about their possible uses in the classroom. LLMs, as AI systems leveraging natural language processing capabilities, can comprehend and generate human language. This feature makes them appropriate for various learning tasks, such as feedback mechanisms, individualized instruction, and automated question-answering systems ( Zhang et al., 2024). One essential use of large language model (LLM) technology in the field of education is personalized learning. LLMs can optimize educational outcomes by creating customized learning trajectories and recommending resources based on students’ interests and progress ( Zhang et al., 2024). Bhutoria (2022) emphasized that LLMs can provide individualized learning recommendations and feedback by analyzing students’ assignments, classroom performance, and learning behaviors. This approach enables learners to acquire knowledge at their own pace. This type of data-driven, individualized instruction improves the learning process overall and significantly increases student autonomy.

The large language models known as LLM-based evaluators are made to evaluate the quality of samples according to predetermined standards. These evaluators can be configured to evaluate individual samples by assigning scores, such as Likert-scale ratings ranging from 1 to 5 ( Likert, 1932), or to compare the quality of paired samples and determine which is superior ( Chiang & Lee, 2023; Zheng et al., 2023). Following the demonstrated capabilities of proprietary LLMs, including ChatGPT, GPT-4, and Claude ( Chiang et al., 2024), as robust LLM-based evaluators ( Chiang & Lee, 2023; Liu et al., 2023; Zheng et al., 2023), subsequent research has focused on enhancing the alignment of their scoring with human evaluators ( Chiang & Lee, 2023; Yuan et al., 2023). Concurrently, another stream of research seeks to improve the performance of open-source LLMs, such as Llama ( Zheng et al., 2023) and Mistral ( Jiang et al., 2023), as evaluators by developing specialized training datasets and employing fine-tuning techniques ( Kim et al., 2023; Li et al., 2023; Kim et al., 2024). Most studies investigating the efficacy of LLMs as automated graders utilize datasets containing student submissions alongside human-assigned scores to evaluate whether LLM-generated assessments align with those of human evaluators ( Latif & Zhai, 2024; Lee et al., 2024). These studies generally operate in controlled settings where LLM-generated scores do not influence students’ grades, and direct interaction between students and the LLMs is absent.

### 2.2 GenAI in education

Generative Artificial Intelligence (GenAI) has become widely available since the launch of ChatGPT in late 2022, affecting both students and teachers in education. The term “GenAI” describes sophisticated AI systems that can predict the next piece of content and produce outputs that resemble those of a person, including text, photos, speech, music, video, and more ( Tillmanns et al., 2025). For instance, Large Language Models (LLMs) like ChatGPT produce text by predicting the most likely next word in a sequence, enabling outputs that often appear plausible but are not inherently accurate ( Cascella et al., 2023; Fui-Hoon Nah et al., 2023). Beyond ChatGPT, other GenAI tools, such as Claude, Gemini, DALL-E, and MidJourney, have gained popularity and accessibility, offering diverse applications across educational contexts. Both students and teachers stand to gain from the revolutionary potential that GenAI presents for education. Students can use GenAI for brainstorming ideas, drafting essays, simulating real-world scenarios, clarifying complex concepts, generating practice exam questions, and receiving personalized feedback ( Atlas, 2023; Fui-Hoon Nah et al., 2023). Time-consuming chores for lecturers, like developing lesson plans, producing practice questions, and giving students tailored answers, are greatly streamlined by GenAI. Teachers can spend more time improving student comprehension and creating more in-depth learning experiences because of these efficiencies ( Ivanov, 2023). While GenAI provides significant benefits, its integration into education also raises ethical concerns. Numerous concerns have been covered, including plagiarism, the spread of false information, bias in AI outputs, and issues with the validity of distant assessments ( Wu & Zhang, 2023). These challenges underscore the importance of educating both students and lecturers on the responsible use of GenAI tools. Notably, banning GenAI entirely, as some higher education institutions initially attempted, has proven difficult to enforce and counterproductive, highlighting the need for guidance and structured policies for its responsible adoption ( Eke, 2023).

### 2.3 In-Service Teachers and Generative AI (GenAI)

Teachers’ perceptions of Generative AI (GenAI) are shaped by their familiarity with the technology, their understanding of its capabilities, and their beliefs regarding its potential benefits and risks. Studies indicate that educators who view GenAI as a tool to enhance instructional practices are more likely to adopt it in the classroom ( Kaplan-Rakowski et al., 2023). Positive perceptions often arise from recognizing GenAI’s capacity to personalize learning experiences, alleviate administrative burdens, and provide innovative teaching resources. For instance, Collie and Martin (2024) observed that teachers’ engagement with GenAI is significantly influenced by factors such as their perception of autonomy-supportive leadership, a commitment to professional growth, and stress associated with adapting to technological changes. Despite these benefits, significant concerns persist. Some educators view GenAI as a potential threat to their professional roles, fearing that it may replace specific teaching functions or reduce the demand for human educators. Ethical concerns further exacerbate resistance, with apprehensions about biased outputs, pseudo-bias, a decline in originality, and issues related to data privacy and security ( Zhai & Krajcik, 2024). Such challenges contribute to skepticism, particularly among educators with limited exposure to or understanding of GenAI’s applications.

### 2.4 Anchored study’s theory

The Technology Acceptance Model (TAM), developed by Davis (1989), serves as the theoretical framework for this study. TAM is a widely recognized model for understanding technology adoption and use, particularly in educational contexts. It posits that two primary determinants influence an individual’s behavioral intention (BI) to adopt a technology: Perceived Usefulness (PU) and Perceived Ease of Use (PEU). Perceived Usefulness refers to the degree to which an individual believes that using a technology will enhance their performance, while Perceived Ease of Use reflects the extent to which a person finds the technology easy to use and free of effort. These constructs are mediated by an individual’s attitude toward the technology (AT), which directly impacts their behavioral intention to adopt it. TAM is particularly well-suited to this study as it aligns closely with the variables under investigation. For instance, Perceived Ease of Use (PEU) in this study corresponds directly to PEOU in TAM, as it examines the extent to which Nigerian in-service teachers perceive ChatGPT as user-friendly and easy to integrate into their teaching practices. Similarly, Perceived Usefulness (PUC) aligns with PU in TAM, representing the teachers’ belief that ChatGPT can enhance their teaching efficiency, support instructional quality, and streamline administrative tasks.

The construct of Attitude Toward ChatGPT (ATC) also maps onto TAM’s focus on user attitudes, capturing teachers’ overall positive or negative feelings about using ChatGPT in their classrooms. Furthermore, Behavioral Intention (BI) in this study is directly derived from TAM and measures teachers’ willingness and intention to adopt ChatGPT as an educational tool. To account for the unique challenges of adopting AI in resource-constrained environments, we extend TAM by incorporating additional variables. For example, Your Colleagues and Your Use of ChatGPT (YCC) reflects the role of social factors in shaping attitudes and behaviors, which is particularly relevant in collaborative teaching environments. Teachers’ Trust in ChatGPT (TTC) introduces the dimension of trust, reflecting teachers’ confidence in ChatGPT’s reliability, accuracy, and ethical considerations. These extensions align with findings from previous research that highlight the importance of trust in adopting new technologies ( Gefen et al., 2003). We also examine Technology Anxiety (TA) and Privacy Issues (PIU) as external variables that may influence PEOU and BI. Technology Anxiety captures the fear or discomfort associated with using AI-based tools, which has been shown to negatively affect technology adoption ( Venkatesh et al., 2003). Similarly, Privacy Issues represent concerns about data security and ethical use, which may diminish teachers’ confidence in ChatGPT and hinder its adoption.

By integrating these constructs, TAM provides a robust framework for exploring both the linear and nonlinear relationships among the variables in this study. The model not only explains how teachers perceive and evaluate ChatGPT but also highlights the contextual barriers that influence their readiness and intention to adopt this technology. This approach ensures a comprehensive analysis of the factors shaping teachers’ behavioral intentions, particularly in resource-constrained environments like Nigeria. The choice of TAM is further supported by its widespread application in educational settings. Studies investigating the adoption of digital classrooms, learning management systems, and AI-based platforms have demonstrated its relevance in capturing user acceptance and identifying key drivers of technology use ( Davis, 1989; Venkatesh & Davis, 2000). Additionally, TAM’s flexibility allows for the integration of external variables, enabling us to adapt the framework to the unique context of ChatGPT adoption in Nigeria.

### 2.5 Hypothesis development

Based on the identified constructs and prior research, the following hypotheses are proposed:

Perceived Ease of Use (PEU) is the degree to which a person thinks a certain technology must be used with little effort. It is a key component of the Technology Acceptance Model (TAM), which holds that consumers’ attitudes and intentions to accept technology are significantly influenced by both perceived utility and PEU ( Misra et al., 2023; Zogheib & Zogheib, 2024). Empirical evidence consistently demonstrates that higher levels of PEU positively influence behavioral intentions to use technologies such as ChatGPT. For example, research conducted among university students revealed that when ChatGPT is perceived as user-friendly, students exhibit a significantly greater intention to adopt it ( Zogheib & Zogheib, 2024; Almogren et al., 2024). These findings align with broader studies underscoring the critical role of both PEU and perceived usefulness in predicting behavioral intentions within educational settings ( Peng & Yan, 2022). In the study by Yang and Wang (2019), perceived ease of use was found to have a significant positive influence on students’ behavioral intentions to adopt machine translation. Similarly, Shiau and Chau (2016) argue that when individuals perceive a technological tool as requiring minimal effort to use, they are more likely to adopt it. Based on these findings, this hypothesis is proposed:

***H1: Perceived Ease of Use (PEU) has a significant positive effect on Behavioral Intention (BI) to adopt ChatGPT.***

Studies have demonstrated a significant positive correlation between perceived usefulness and behavioral intention. For instance, research focusing on doctoral students revealed that when ChatGPT is perceived as beneficial for improving academic writing, students exhibit a substantially higher intention to adopt it ( Zou & Huang, 2023). This finding aligns with the core principles of the Technology Acceptance Model (TAM), which identifies perceived usefulness as a key determinant of technology adoption ( Yang & Wang, 2019). Accordingly, the following hypothesis is proposed:

***H2: Perceived Usefulness (PUC) has a significant positive effect on Behavioral Intention (BI) to adopt ChatGPT.***

Attitude refers to the psychological system individuals use to evaluate the effects of a particular act, encompassing beliefs, emotions, and behaviors toward an object, event, or person. This directly influences behavioral intentions to take action ( Athiyaman, 2002; Davis, 1989). Within the frameworks of the Technology Acceptance Model (TAM) and the Theory of Reasoned Action ( Fishbein & Ajzen, 1977), attitude has been extensively studied. Students’ attitudes toward using ChatGPT for educational purposes can vary, ranging from positive to negative. In TAM, perceived ease of use significantly impacts perceived usefulness and attitudes, which in turn strengthens behavioral intention ( Davis, 1989; Alfadda & Mahdi, 2021). When a technological tool is perceived as easy to use, students are more likely to find it helpful, develop a positive attitude, and show a strong willingness to utilize it, particularly in academic writing ( Alfadda & Mahdi, 2021). Consequently, this study hypothesizes that attitudes toward using ChatGPT for learning significantly influence their behavioral intentions, leading to the following hypothesis.

***H3: Attitude Toward ChatGPT (ATC) has a significant positive effect on Behavioral Intention (BI) to adopt ChatGPT.***

Research demonstrates that higher levels of trust in ChatGPT significantly increase teachers’ behavioral intentions to adopt the technology. Studies show that when educators perceive ChatGPT as a reliable and trustworthy resource, they are more inclined to integrate it into their teaching practices ( Bhaskar et al., 2024; Bhat et al., 2024). Trust also serves as a moderating factor in the relationship between perceived usefulness and behavioral intention. When teachers trust ChatGPT, their confidence in its capabilities enhances their perception of its usefulness, which in turn strengthens their intention to utilize it ( Bhaskar et al., 2024). Moreover, teachers who trust the technology are more likely to incorporate it consistently into their classrooms, reinforcing their initial intentions to use it ( Choudhury & Shamszare, 2023). Supporting this, a survey of 359 participants found that users’ trust in chatbot services significantly influenced their intention to continue using them ( Nguyen et al., 2021). These findings underscore the critical role of trust in the adoption and implementation of ChatGPT, affirming its positive impact on actual usage. Based on this, the following hypothesis was formulated:

***H4: Teachers’ Trust in ChatGPT (TTC) has a significant positive effect on Behavioral Intention (BI) to adopt ChatGPT.***

Technology anxiety refers to the apprehension or fear individuals experience when interacting with technology, often stemming from concerns about its usability, reliability, or potential negative outcomes ( Budhathoki et al., 2024). Research consistently shows that higher levels of technology anxiety are associated with lower behavioral intentions to adopt ChatGPT. Studies applying the Unified Theory of Acceptance and Use of Technology (UTAUT) framework found that anxiety significantly negatively impacted students’ intentions to use ChatGPT in educational contexts across diverse regions, including the UK and Nepal ( Budhathoki et al., 2024). Additionally, research on the moderated mediation model of technology anxiety highlights its multifaceted impact. Technology anxiety not only directly influences behavioral intention but also interacts with other factors, such as technostress, exacerbating stress levels associated with using ChatGPT. This heightened stress further reduces the likelihood of adopting the technology ( Duong et al., 2024). Based on these findings, the following hypothesis was proposed:

***H5: Technology Anxiety (TA) has a significant negative effect on Behavioral Intention (BI) to adopt ChatGPT.***

Privacy concerns center on how personal data is collected, stored, and utilized by technologies like ChatGPT, often leading to user apprehension. Studies show that perceived privacy risks significantly reduce users’ intentions to adopt such technologies. For instance, unresolved privacy concerns have been found to undermine trust, resulting in reluctance to engage with AI tools perceived as risky in terms of data security ( Wu et al., 2024). When users believe their personal information may be mishandled or insufficiently protected, their willingness to use ChatGPT declines. Based on these insights, the following hypothesis was proposed:

***H6: Privacy Issues (PIU) have a significant negative effect on Behavioral Intention (BI) to adopt ChatGPT.***

The adoption of ChatGPT is significantly influenced by the behavior and attitudes of peers and colleagues. When individuals observe their colleagues using ChatGPT effectively, they are more likely to form a favorable attitude toward the tool and develop an intention to adopt it themselves. Research highlights that positive word-of-mouth and peer recommendations play a crucial role in enhancing users’ adoption intentions ( Al-Kfairy, 2024; Jo & Bang, 2023). Observing peers benefit from the technology reinforces an individual’s belief in its usefulness, further increasing their likelihood of engagement ( Al-Kfairy, 2024). The Theory of Planned Behavior underscores the role of subjective norms, which are shaped by the behaviors and expectations of colleagues, in influencing individual attitudes toward adopting new technologies. When individuals perceive a social expectation to use ChatGPT, their intention to adopt it is strengthened ( Al-Kfairy, 2024). Based on these findings, the following hypothesis was proposed:

***H7: Your Colleagues and Your Use of ChatGPT (YCC) have a significant positive effect on Behavioral Intention (BI) to adopt ChatGPT.***

### 2.6 Literature gap, and study objective

Despite the growing integration of GenAI, formalized frameworks for its ethical and practical use in education remain underdeveloped. Unlike the generally accepted principles for robotics, such as Asimov’s Three Laws ( Asimov, 2004), there are no universally established guidelines for GenAI in education. Previous literature reviews have explored topics such as the role of ChatGPT in higher education ( Bhullar et al., 2024), AI chatbots ( Labadze et al., 2023), and ethical principles ( Nguyen et al., 2023a). However, limited research has explored the integration of LLMs into real-world classroom environments where students can interact with these evaluative tools ( Nilsson & Tuvstedt, 2023). Consequently, the impact of LLM-based evaluators on teachers’ experiences and perceptions within authentic educational contexts remains largely underexplored. Addressing these concerns is crucial to fostering wider acceptance and integration of GenAI in education.

## 3. Methods

### 3.1 Research context and participants

Our study examines the in-service teachers’ behavioral intentions to use GenAI in their teaching practices. A total of 260 in-service teachers participated in the study after receiving training on the applications of AI in education (AIED). While a majority of the teachers are not new to AIED, yet they further received trainings on the overview of AI concepts, its educational applications, and practical tools for integrating AI technologies into classroom environments. Thereafter, the teachers were invited to willingly participate in completing a standardized survey designed using Google Forms (Google LLC, Mountain View, CA, USA) to capture their insights on the adoption of AI in education. The Google Forms-based survey was distributed through teachers’ professional platforms, specifically WhatsApp (Meta Platforms, Inc., Menlo Park, CA, USA), ensuring easy access for interested participants. This approach facilitated data collection from a broad and diverse sample of educators, allowing them to confidentially share their experiences and expectations regarding AI tools in education. Table 1 below shows the demographic profile of the 260 respondents who participated in the survey.

**Table 1.** Demographic profile of the respondents (N =260).

Variable	Categories	Frequency	Percent
Gender	Male	111	42.7
	Female	149	57.3
Age group	Less than or equal to 25 years	4	1.5
	26-29 years	14	5.4
	30-39 years	109	41.9
	40-49 years	100	38.5
	50+ years	33	12.7
Subject area	Science & Technology	153	58.8
	Arts/Humanities	30	11.5
	Others	77	29.6
School type	Public	154	59.2
	Private	106	40.8
School location	Rural	38	14.6
	Urban	172	66.2
	Semi-urban	50	19.2
Technology usage competency level	Novice	6	2.3
	Beginner	22	8.5
	Intermediate	145	55.8
	Advance	87	33.5

(Source: table created by authors).

Table 1 provides a comprehensive overview of the demographic profile of our respondents, detailing their gender, age, subject area, school type, school location, and technology usage competency level. Our results reveal that most respondents are female, with male participants forming a smaller proportion of the sample. Most respondents are mid-career professionals, predominantly between 30 and 49, while younger and older teachers are less represented. Regarding subject specialization, a significant portion of the respondents teach Science and Technology-related subjects, while a smaller proportion specialize in Arts, Humanities, and other disciplines. Regarding school type, more teachers are employed in public schools, although private school teachers also constitute a notable segment, ensuring balanced representation from both educational settings. Our results further indicate that most respondents work in urban areas, followed by those in semi-urban and rural locations. This suggests that technology exposure and accessibility may be higher in urban settings. Additionally, most participants demonstrate a strong level of digital competence, with most identifying as intermediate or advanced technology users. Only a small fraction considers themselves beginners or novices, highlighting teachers’ overall familiarity and confidence in engaging with digital tools in their professional practice.

### 3.2 Study instrument

The data for this study were collected from in-service teachers through a Google Forms-based survey distributed across their professional platforms. The survey was adapted from the works of Ayanwale et al. (2022) and Nazaretsky et al. (2021) to align with the specific objectives of this research. The constructs related to trust were adapted from Nazaretsky et al. (2021), while other constructs were drawn from Ayanwale et al. (2022). Additional items were incorporated into the final instrument to better suit the context of our study. Prior to administration, the survey underwent a reliability test (refer to Table 2 for indicator reliability summary). The final version of the instrument consisted of nine sections, which were reviewed and validated by experts in the fields of AI in education (AIED), Test and Measurement, and Educational Technology. The first section of the survey gathered demographic information from participants, including gender, age, subject taught, school type, school location, and ICT competency level. The remaining eight sections measured the key constructs – perceived usefulness of ChatGPT (PUC), perceived ease of use of ChatGPT (PEU), teachers’ trust in ChatGPT (TTC), privacy issues and use of ChatGPT (PIU), your colleagues and your use of ChatGPT (YCC), technology [ChatGPT] anxiety (TA), attitude towards ChatGPT (ATC), and behavioural intention to use ChatGPT (BI) – of the study. The 45-item survey was then distributed online to the teachers, with responses rated on a Likert scale ranging from “strongly disagree” (1) to “strongly agree” (6). The survey remained open for data collection for two months, during which daily reminders were sent across professional platforms to encourage voluntary participation. After the two-month period, the survey was closed, and no further responses were accepted. This approach allowed us to gather a diverse range of responses while ensuring that all participants had equal opportunity to contribute.

**Table 2.** Summary of indicator reliability.

Indicators	Item loadings	Standard deviation	T statistics	P values
ATC1 <- ATC	0.946	0.031	30.316	0.000
ATC2 <- ATC	0.970	0.037	26.141	0.000
ATC3 <- ATC	0.978	0.032	30.823	0.000
BI1 <- BI	0.947	0.011	87.446	0.000
BI2 <- BI	0.957	0.008	123.733	0.000
BI3 <- BI	0.933	0.012	75.329	0.000
BI4 <- BI	0.933	0.011	88.628	0.000
PEU1 <- PEU	0.935	0.043	21.885	0.000
PEU2 <- PEU	0.948	0.043	22.260	0.000
PEU3 <- PEU	0.887	0.076	11.653	0.000
PEU4 <- PEU	0.902	0.072	12.512	0.000
PEU5 <- PEU	0.929	0.055	16.771	0.000
PEU6 <- PEU	0.962	0.046	21.040	0.000
PIU1 <- PIU	0.982	0.022	44.282	0.000
PIU2 <- PIU	0.977	0.025	39.184	0.000
PIU3 <- PIU	0.974	0.021	46.649	0.000
PUC1 <- PUC	0.912	0.017	53.004	0.000
PUC2 <- PUC	0.910	0.021	42.728	0.000
PUC3 <- PUC	0.902	0.025	35.416	0.000
PUC4 <- PUC	0.894	0.020	44.222	0.000
PUC5 <- PUC	0.878	0.020	43.358	0.000
PUC6 <- PUC	0.895	0.013	68.691	0.000
TA1 <- TA	0.943	0.051	18.444	0.000
TA2 <- TA	0.943	0.057	16.690	0.000
TA3 <- TA	0.975	0.054	18.128	0.000
TA4 <- TA	0.973	0.052	18.750	0.000
TTC1 <- TTC	0.954	0.135	7.078	0.000
TTC2 <- TTC	0.961	0.134	7.187	0.000
TTC3 <- TTC	0.965	0.131	7.381	0.000
TTC4 <- TTC	0.913	0.147	6.194	0.000
YCC1 <- YCC	0.939	0.019	48.691	0.000
YCC2 <- YCC	0.985	0.018	55.933	0.000
YCC3 <- YCC	0.977	0.021	46.533	0.000
YCC4 <- YCC	0.983	0.018	54.183	0.000

Note: PEU **-** Perceived Ease of Use, PUC - Perceived Usefulness, ATC - Attitude Toward ChatGPT, TTC - Teachers’ Trust in ChatGPT, TA - Technology Anxiety, PIU - Privacy Issues, YCC - Your Colleagues and Your Use of ChatGPT

(Source: table created by authors)

### 3.3 Content validity

To ensure the content validity of the survey instrument, the final version was reviewed and validated by experts in the fields of AI in education (AIED), Test and Measurement, and Educational Technology. This ensured that the instrument accurately represents the construct it is intended to measure. The goal of our study was to capture in-service teachers’ perspectives and intention to integration of AI tools and the survey was designed to achieve this goal. The validation process involved expert reviews where each item was scrutinized for relevance, clarity, and alignment with the research objectives. The experts also provided feedback on the wording of certain questions to ensure they were accessible to a diverse population of teachers with varying levels of expertise in AI. Thereafter, the final instrument was considered both comprehensive and contextually appropriate for capturing the data necessary to address the hypotheses raised in this study.

### 3.4 Method of data collection

The data for this study ( Ayanwale et al., 2025) were collected through a Google Forms-based survey, which was shared with participants via teachers’ professional platforms, particularly WhatsApp. This method of distribution ensured that the survey was easily accessible to a wide audience of educators who were part of professional networks. Participants were provided with clear instructions and were required to give informed consent before completing the survey online. Basically, by accepting to fill the survey, a teacher accepts to willingly participate in the study. This was crucial to ensure participants understood the purpose of the study, the voluntary nature of their participation, and the confidentiality of their responses, the information of which was provided along with the survey online. By using professional platforms like WhatsApp, we were able to reach a broad and diverse sample of teachers, including those from varying geographic locations, subject areas, and teaching levels. This approach also enabled participants to share their experiences and expectations regarding AI tools in education in a confidential and comfortable setting. The survey was structured to be straightforward, allowing teachers to respond based on their own experiences and perspectives on AI adoption. The survey remained open for a period of two months to allow ample time for participation. After the two-month data collection window, the survey was closed, and no further responses were accepted.

### 3.5 Method of data analysis

In our study, we employed a dual-stage approach that integrated Partial Least Squares Structural Equation Modeling (PLS-SEM) with Artificial Neural Networks (ANN) to predict Nigerian in-service teachers’ behavioral intention (BI) to adopt ChatGPT. This hybrid methodology allowed us to leverage the strengths of both techniques: PLS-SEM offered insights into linear relationships and supported theory development, while ANN uncovered complex, nonlinear interactions and ranked the importance of predictors. Together, these methods provided a comprehensive understanding of the key drivers and barriers to ChatGPT adoption. We chose PLS-SEM for its effectiveness in predictive research, particularly with small sample sizes, as noted by Hair et al. (2021), who affirm its reliability with as few as five cases per parameter. PLS-SEM also adeptly handles non-normal data distributions, making it well-suited for our dataset. Using the seminr and plspm packages ( Hair et al. 2021) in RStudio (version 4.3.3) ( R Core Team, 2021), we specified the measurement and structural models. Constructs were defined as reflective, and we assessed their reliability and validity using Cronbach’s alpha, composite reliability, and Average Variance Extracted (AVE). We confirmed discriminant validity through the Heterotrait-Monotrait (HTMT) ratio. The structural model was analyzed to estimate path coefficients and their significance using bootstrapping with 10,000 resamples. Additionally, we evaluated model fit using the Standardized Root Mean Residual (SRMR) and, calculated R ^2^ values to measure the explained variance in BI, f ^2^ effect sizes for predictors, and Q ^2^ values via PLSpredict to assess predictive relevance.

**Figure 1.**
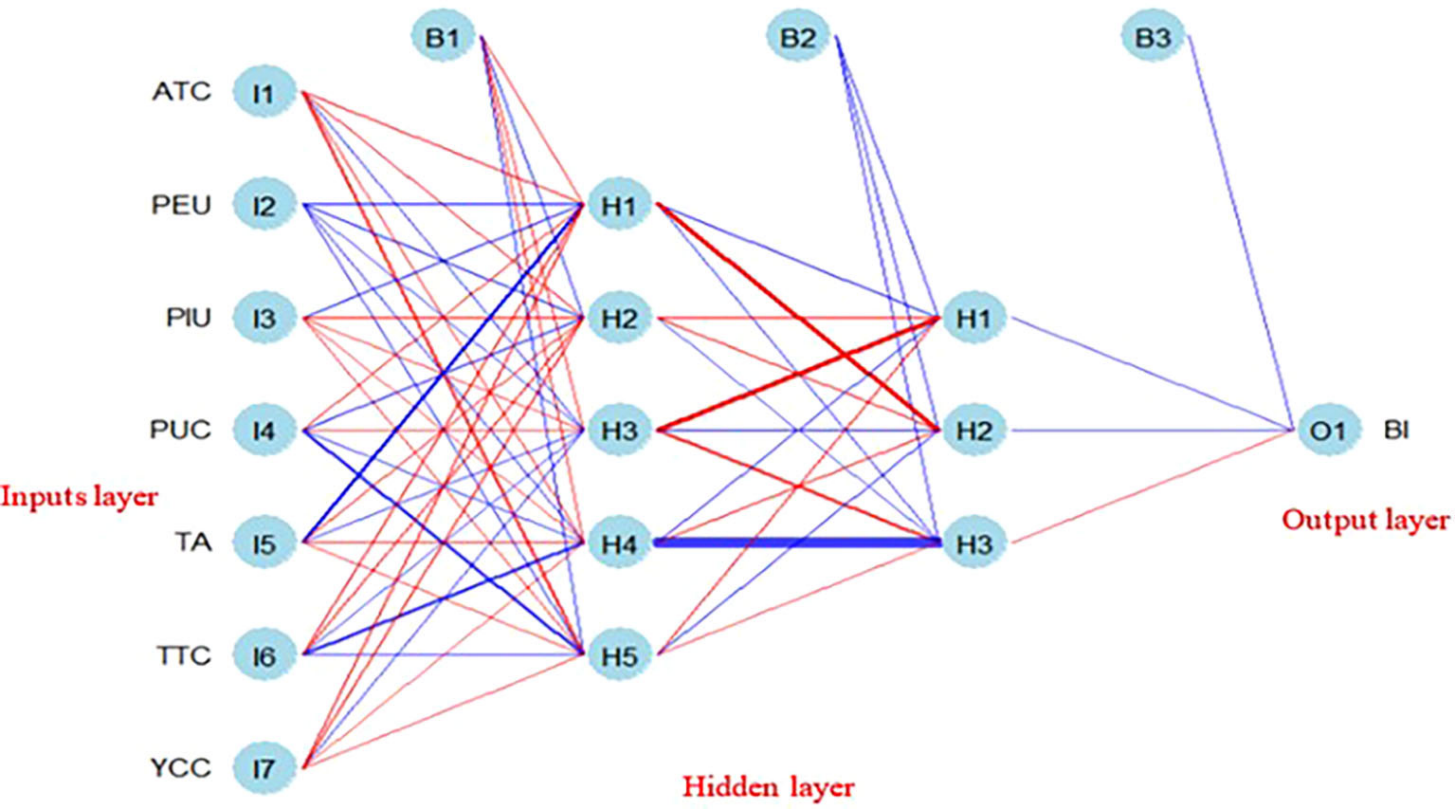
Illustration of how the backpropagation works by adjustments of weights. (Source: figure created by authors).

To complement the linear insights from PLS-SEM, we employed ANN to investigate nonlinear relationships using the neuralnet and NeuralNetTools packages. Our ANN model was trained with a backpropagation algorithm, specifically through feed-forward networks, which adjusted weights iteratively to minimize prediction errors. The model architecture comprised an input layer, two hidden layers with five and three neurons, respectively, and an output layer (see Figure 1). We assessed the model’s predictive performance through Root Mean Squared Error (RMSE) and Mean Absolute Error (MAE), confirming its high accuracy. We also utilized Garson’s and Olden’s algorithms to rank the importance of predictors, identifying key factors such as PEU, PUC, and ATC as critical determinants. This analysis validated the linear results from PLS-SEM and offered additional insights into the nonlinear dynamics influencing BI. By integrating PLS-SEM and ANN, we not only confirmed our model’s findings but also deepened our understanding of the factors influencing teachers’ readiness to adopt ChatGPT. This combined approach aligns with previous research highlighting the complementary strengths of SEM and ANN in predictive studies ( Hair et al., 2021; Leong et al., 2024). The detailed R codes used in our analysis are provided in the supplementary file for transparency and replicability.

### 3.8 Ethical consideration

In our study, we emphasized ethical considerations to ensure the integrity of our research process. Ethical clearance was obtained from the Department of Counseling Psychology and Educational Foundation Ethical Review Board at Tai Solarin University of Education, with approval granted on July 22, 2024, under Ethical Clearance Number ECN 07-2024-15. This approval confirmed our adherence to established ethical standards for studies involving human participants and aligned with the principles outlined in the Declaration of Helsinki, which governs research involving human subjects. To uphold these ethical standards, we ensured that respondents were fully informed about the purpose of the research, the voluntary nature of their participation, and the confidentiality of their responses. This information was provided alongside the survey, which was shared on professional teachers’ platforms. Respondents could not proceed to complete the online form without first reading and accepting this information. This process guaranteed that written informed consent was obtained electronically before participation, reinforcing their right to decide voluntarily about their involvement. Additionally, we maintained strict confidentiality throughout the study. Responses were securely stored in password-protected digital files and were accessible only to the research team, safeguarding participant privacy. By completing the survey, respondents willingly agreed to participate, fully aware of their rights and the protective measures in place. These ethical commitments underscore our dedication to responsible research practices and the ethical integrity of our study.

## 4. Results

We present the results highlighting key predictors of teachers’ behavioral intention to adopt ChatGPT, based on a hybrid approach that combines PLS-SEM and ANN analyses.

### 4.1 Model fit assessment

We established a model fit assessment that demonstrates our structural model fits the data well. The SRMR value of 0.049, well below the threshold of 0.08, indicates excellent alignment between observed and predicted correlations ( Hair et al., 2021). Both the Unweighted Least Squares Discrepancy (d_ULS) value of 1.419 and the Geodesic Discrepancy (d_G) value of 7.718 reflect minimal discrepancies, further supporting the model’s adequacy. Although the chi-square value of 6774.515 is influenced by sample size and model complexity, we find it reasonable in context. The NFI value of 0.880, nearing the recommended threshold of 0.90, suggests an acceptable model fit. Overall, these results confirm that our model provides a solid foundation for analyzing relationships and validates our study.

### 4.2 Evaluation of the measurement model in PLS-SEM

In our PLS-SEM analysis, we evaluated the measurement model to ensure that the constructs used in the study met the necessary reliability and validity benchmarks. This assessment focused on three key areas: reliability, convergent validity, and discriminant validity, which are essential for establishing the robustness and accuracy of the structural model results.

**Figure 2.**
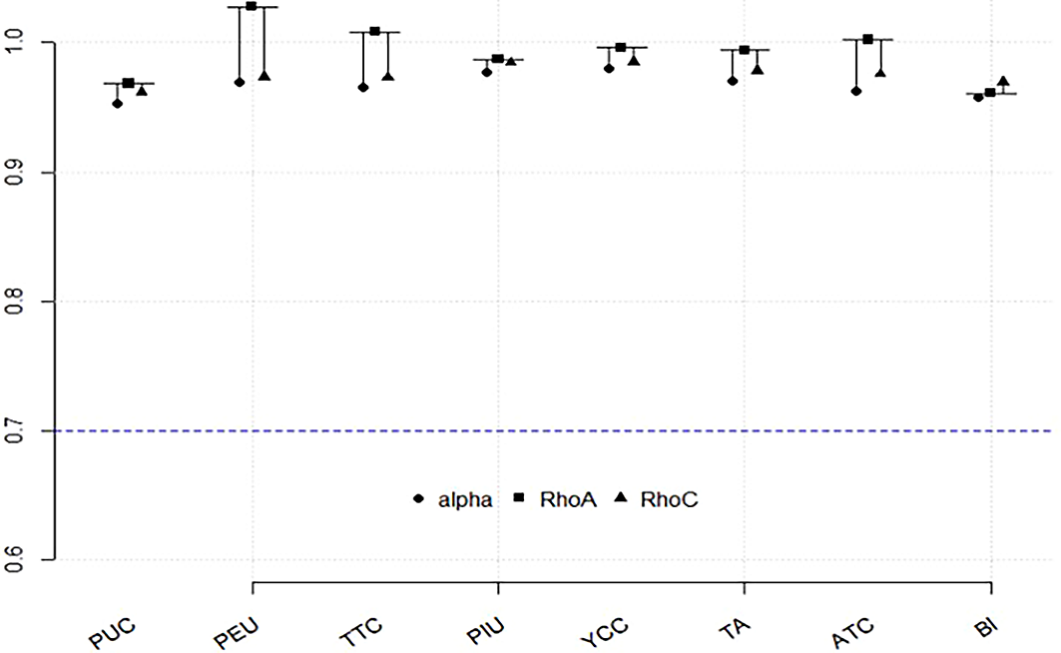
Reliability and validity of the measurement model. (Source: figure created by authors).

To assess reliability, we examined indicator reliability, Cronbach’s alpha, and composite reliability coefficients (rho_c). These measures indicate the internal consistency of the constructs, ensuring that the indicators within each construct consistently measure the underlying variable. According to Hair et al. (2019, 2021), a threshold of 0.70 is recommended for all three metrics. In our study, indicator reliability values ranged from 0.878 to 0.985 (see Table 2), while Cronbach’s alpha values ranged from 0.953 to 0.980, and rho_c values ranged from 0.962 to 0.985 (see Figure 2). These results confirm that all constructs in the model were well above the thresholds and demonstrated strong internal consistency, highlighting the reliability of the latent variables used. Convergent validity was evaluated to determine whether the indicators of each latent construct adequately explain its variance. Following Hair et al. (2022), and Magno et al. (2024), we used the average variance extracted (AVE) as the primary metric for this assessment, with a recommended threshold of 0.50. In our study, AVE values ranged from 0.807 to 0.943, well above the benchmark (see Figure 2).

Furthermore, we assessed discriminant validity using the heterotrait-monotrait ratio (HTMT) proposed by Henseler et al. (2015) to ensure that the constructs were distinct and measured their intended concepts. Discriminant validity ensures that a construct is empirically distinct from other constructs in the model. For HTMT, a value below 0.85 (or 0.90 in specific contexts) is considered acceptable ( Henseler et al., 2015). In our analysis, all HTMT values (see Table 3) fell below the threshold of 0.85, confirming that our constructs were empirically distinct from one another.

**Table 3.** Discriminant validity – Heterotrait mono trait ratio.

Constructs	ATC	BI	PEU	PIU	PUC	TA	TTC	YCC
ATC								
BI	0.193							
PEU	0.044	0.201						
PIU	0.017	0.225	0.058					
PUC	0.206	0.296	0.083	0.557				
TA	0.081	0.174	0.257	0.239	0.435			
TTC	0.406	0.108	0.302	0.684	0.764	0.226		
YCC	0.443	0.260	0.156	0.166	0.359	0.154	0.484	

(Source: table created by authors)

### 4.3 Evaluation of the structural model in PLS-SEM

After confirming the reliability and validity of the measurement model, we evaluated the structural model to test the hypothesized relationships among constructs and assess the model’s predictive power. This evaluation included analyzing path coefficients, R ^2^ values, f ^2^ effect sizes, and predictive relevance using PLSpredict. Path coefficients, which represent the strength and direction of relationships between constructs, were assessed using a bootstrapping procedure with 10,000 resamples to determine significance levels (see Table 4). In our study, PUC, TA, YCC, and PEU showed significant relationships with BI. PUC had the most potent positive effect (β=0.879, p<0.05), emphasizing its critical role in predicting teachers’ intention to adopt ChatGPT. Conversely, TA and PEU significantly negatively affected BI (β=−0.548, p<0.05; β=−0.451, p<0.05), indicating that higher anxiety levels reduce behavioral intention. Meanwhile, YCC (β=0.546, p<0.05) contributed positively, underscoring the influence of peer support in adoption decisions. The coefficient of determination (R ^2^), which measures the variance in the dependent variable explained by the independent variables, was 0.158 for BI, indicating that 15.8% of the variance in BI was explained by the model. While moderate, this value reflects the exploratory nature of our study.

**Table 4.** Summary of direct effects relationship in the structural model.

Relationships	β	T-Statistic	P-value	Interpretation
PUC -> BI	0.879	3.959	0.000	Strong positive effect (significant)
TA -> BI	-0.548	2.402	0.008	Strong negative effect (significant)
YCC -> BI	0.546	2.228	0.013	Moderate positive effect (significant)
PEU -> BI	-0.451	1.937	0.026	Moderate negative effect (significant)
TTC -> BI	-0.262	1.561	0.059	Weak negative effect (not significant)
ATC -> BI	0.244	1.404	0.080	Weak positive effect (not significant)
PIU -> BI	-0.179	0.905	0.183	Weak negative effect (not significant)
Predictor	Effect Size (f ^2^)			
PEU	0.173 (Moderate)			
PUC	0.274 (Large)			
TA	0.220 (Moderate)			
TTC	0.216 (Moderate)			
YCC	0.023 (Small)			
ATC	0.006 (Negligible)			
PIU	0.003 (Negligible)			

Note: one tail-test, t >1.645; (Source: table created by authors).

Additionally, effect sizes (f ^2^) were calculated to evaluate the relative importance of each predictor (see Table 5). Following Cohen (1992) guidelines, f ^2^ values of 0.02, 0.15, and 0.35 indicate small, moderate, and large effects, respectively. In our model, PEU, PUC, TA, and TTC had a moderate effect size, while the other constructs showed small or negligible effects. To assess predictive relevance, we employed PLSpredict, which evaluates the model’s performance in predicting new observations. The Q ^2^ value, derived through PLSpredict, was 0.137 for BI, confirming the model’s predictive relevance. This value demonstrates that the model can make meaningful out-of-sample predictions, an essential feature for practical application and validation.

**Table 5.** Summary of ANN ranked variables and their weights.

Rank	Variable	Weight	Importance
1	PEU	107.416	Most important
2	ATC	44.349	High importance
3	PUC	31.012	Moderate importance
4	YCC	16.386	Moderate importance
5	TTC	-7.227	Low importance
6	TA	-67.340	Very low importance
7	PIU	-157.447	Least important

(Source: table created by authors).

### 4.4 Artificial Neural Network analysis

To complement our findings from the PLS-SEM analysis and to uncover complex nonlinear relationships among variables, we employed Artificial Neural Network (ANN) analysis. Initially, we used PLS-SEM to examine the hypothesized relationships and to identify key factors influencing in-service teachers’ behavioral intention (BI) to adopt ChatGPT in their classrooms. In the second phase, we utilized ANN analysis to rank the predictors affecting BI and to provide deeper insights into the adoption dynamics. Following the methodology outlined by Liébana-Cabanillas et al. (2017), we integrated the PLS-SEM latent scores as neural inputs for the ANN model. This dual-stage approach enabled us to validate the statistical significance of predictors while capturing intricate nonlinear interactions. Prior to training the ANN model, we conducted data preprocessing to ensure compatibility. This involved normalizing all variables using min-max scaling, which transformed values into a range of 0 to 1 to prevent variables with larger scales from exerting disproportionate influence. The dataset was subsequently split into a training set (70%) and a testing set (30%), in line with Leong et al. (2024), ensuring effective learning while maintaining generalizability. To mitigate overfitting, we implemented a ten-fold cross-validation procedure, as recommended by Ooi and Tan (2016), allowing us to obtain the Root Mean Squared Error (RMSE) and enhance predictive accuracy.

The ANN model comprised an input layer with seven neurons corresponding to the independent variables: PEU, PUC, ATC, TTC, TA, PIU, and YCC. A multilayer perceptron architecture was adopted, incorporating two hidden layers with five and three neurons, respectively. These configurations were determined heuristically to balance computational efficiency with predictive capability. The output layer contained a single neuron representing the dependent variable, BI. Model training was conducted using the feed-forward-backward propagation (FFBP) algorithm, which forwards inputs and refines error estimations through backward propagation ( Taneja & Arora, 2019). This iterative learning process allowed the model to continuously adjust weights, enhancing prediction accuracy. The training phase involved a forward pass, during which input data was processed through the network, followed by error calculation using Mean Squared Error (MSE). A backward pass then optimized the weights through gradient descent, ensuring the model learned from past errors and improved accuracy with each iteration. Sigmoid activation functions were employed in both the input and hidden layers to introduce nonlinearity, thereby enabling the model to capture intricate relationships. The training process continued until errors were minimized, further refining the network’s predictive performance.

**Figure 3.**
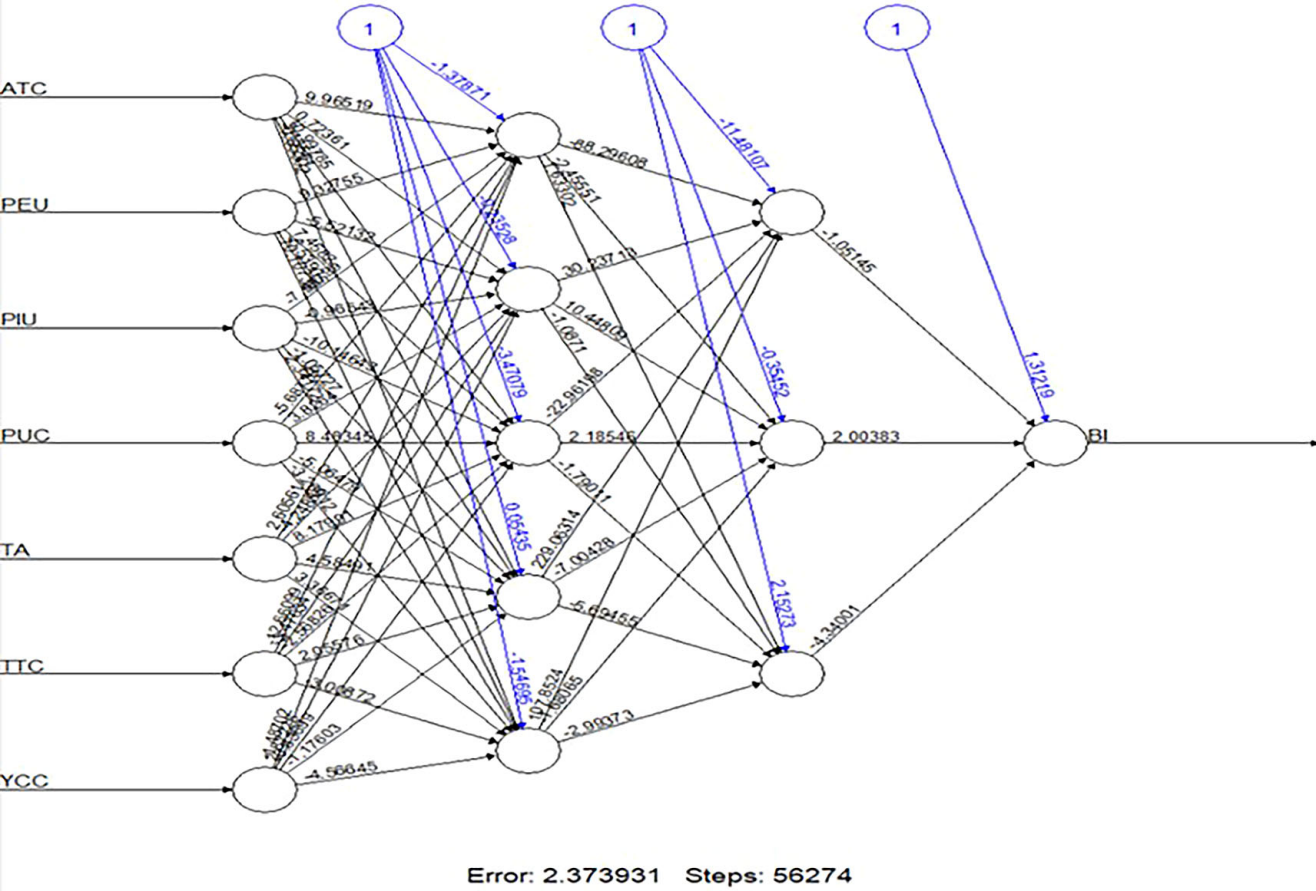
The structure of the ANN highlights the weights assigned to each construct. (Source: figure created by authors).

**Figure 4.**
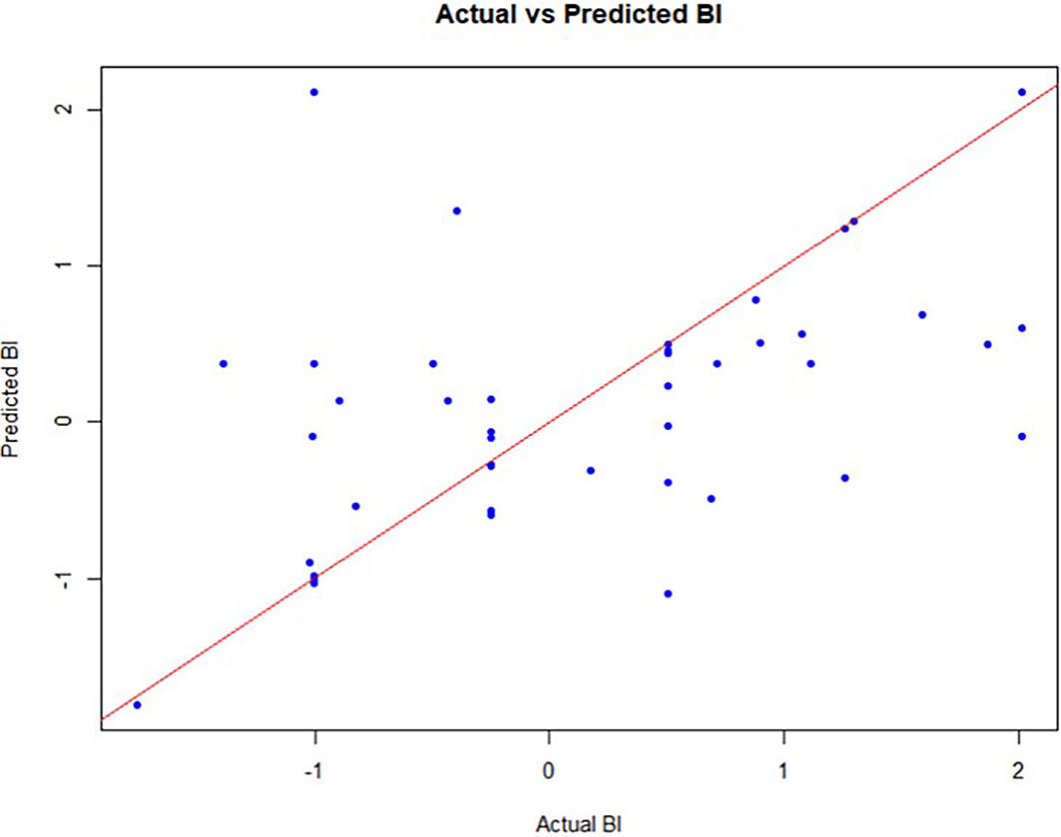
Scatter plot depicts the actual values and the predicted values of BI. (Source: figure created by authors).

We computed key performance metrics to assess the model’s accuracy, including RMSE and Mean Absolute Error (MAE). The ANN model yielded an RMSE value of 0.87, demonstrating strong predictive performance and confirming its effectiveness in identifying patterns within the dataset. Additionally, we employed Garson’s and Olden’s algorithms to analyze connection weights and determine the relative importance of each predictor influencing BI (see Table 6). The results indicated that PEU was the most influential predictor, followed by ATC and PUC, underscoring the critical roles of ease of use, positive attitudes, and perceived benefits in driving ChatGPT adoption. YCC and TTC exhibited moderate influence, whereas TA had a slight but notable negative impact. PIU was identified as the least significant factor, suggesting that privacy concerns did not constitute a major barrier in this context. The ANN model visualization provided further insight into the strength of connections between variables and neurons, reinforcing the ranking of predictor importance (see Figure 3). Additionally, a scatter plot comparing actual versus predicted BI values (see Figure 4) illustrated the model’s ability to produce accurate predictions. The ANN analysis not only validated but also extended the findings from PLS-SEM by uncovering nonlinear relationships and ranking predictors based on their impact. While PLS-SEM confirmed significant linear relationships, ANN revealed additional complexities in the data that traditional regression techniques might overlook.

**Table 6.** Summary of PLS-SEM ranked variables and their weights.

Ranking	Variable importance	Path weights
1	PUC	0.879
2	TA	-0.548
3	YCC	0.546
4	PEU	-0.451
5	TTC	-0.262
6	ATC	0.244
7	PIU	-0.179

(Source: table created by authors)

We also compare the rankings and importance of variables identified by PLS-SEM (see Table 4) and ANN (see Table 5). Both methods offer valuable yet distinct insights into the determinants of behavioral intention (BI). PLS-SEM reveals statistically significant relationships among variables, emphasizing a linear perspective that highlights direct connections between factors and BI. It underscores the importance of PUC and TA, indicating that teachers’ perceptions of usefulness strongly drive adoption, with anxiety acting as a significant deterrent. In contrast, ANN uncovers more complex, nonlinear interactions and prioritizes PEU and ATC as the most critical factors. This suggests that ease of use and attitude are fundamental to adoption, even though their direct effects were weaker in the PLS-SEM analysis. The differing rankings of PUC indicate that while PLS-SEM identifies usefulness as the primary driver, ANN recognizes that usability (PEU) is a crucial first step toward making ChatGPT valuable. Additionally, TA’s lower ranking in the ANN analysis implies that, despite its significant direct effect in PLS-SEM, it may exert indirect or nonlinear influences that ANN does not fully capture.

## 5. Discussions

Our study adopted a hybrid analytical approach combining Partial Least Squares Structural Equation Modeling (PLS-SEM) and Artificial Neural Networks (ANN) to identify key predictors of teachers’ BI to adopt ChatGPT in educational settings. This innovative method allowed us to examine both the linear relationships between variables and more complex, nonlinear interactions which provided a platform to better understand the factors driving AI adoption by in-service teachers in education.

Our results show that notably, PUC emerged as the most influential predictor of BI, with a strong positive effect. This reinforces the idea that teachers are more likely to adopt ChatGPT if they perceive it as useful in their teaching practices ( Alfadda & Mahdi, 2021; Athiyaman, 2002). On the other hand, TA and PEU were found to negatively affect BI ( Wu et al., 2024; Zou & Huang, 2023; Yang & Wang, 2019). These results suggest that anxiety about using the technology and perceived difficulty in operating it can serve as significant barriers to adoption. Social influences, particularly YCC, also played a crucial role, indicating that teachers are more likely to adopt ChatGPT if their colleagues are doing so ( Williams et al., 2014; Wang & Chou, 2014; Venkatesh et al., 2003). Our model’s coefficient of determination for BI was 0.158, meaning that the model explained 15.8% of the variance in teachers’ behavioral intentions to use ChatGPT. Although this value is moderate, it reflects the complexity of real-world adoption processes, where many external factors could influence decisions. On the other hand, effect sizes revealed that PUC had a large effect size, emphasizing its importance, while PEU, TA, and TTC had moderate effects ( Ayanwale et al., 2024; Wu et al., 2024; Zou & Huang, 2023). These findings provide better understanding into the factors having the greatest influence on teachers’ intentions to adopt ChatGPT.

To complement the results from PLS-SEM, our study employed ANN analysis. This allowed for the identification of more intricate, nonlinear relationships among the predictors. The ANN model confirmed that PEU was the most influential factor in predicting teachers’ behavioral intentions to use ChatGPT ( Wu et al., 2024; Zou & Huang, 2023), followed by ATC and PUC. This suggests that, while PLS-SEM identified linear relationships, ANN was able to capture more complex interactions that might not be fully explained by traditional models. Interestingly, while TA had a strong negative effect in PLS-SEM, its importance was much lower in the ANN model, suggesting that its influence could be indirect or dependent on other factors that were not captured in a linear framework. The ANN analysis provided a ranking of the predictors, reinforcing that PEU and ATC were central to teachers’ decisions to adopt ChatGPT, even though their direct effects were weaker in the PLS-SEM analysis. This further show the value of using a hybrid approach to capturing both linear and nonlinear dynamics in technology adoption studies.

Our study’s hybrid approach which combined PLS-SEM and ANN was able to provide a peak into understanding the factors influencing teachers’ behavioral intention to adopt ChatGPT in educational settings. While PLS-SEM validated the significance of linear relationships, ANN revealed the nonlinear complexities underlining the adoption process. Key predictors such as perceived ease of use, perceived usefulness, and attitude were identified as pivotal in the adoption of ChatGPT in education, with social influence and technology anxiety also playing significant roles. This dual-method approach offers a more nuanced perspective on AI adoption in education, emphasizing the importance of both technical factors (ease of use and usefulness) and social considerations (peer influence) in facilitating the integration of AI into teaching practices among teachers. Finally, our results contribute to the ongoing discussion concerning the adoption of AI in educational domains by revealing the complex interplay among cognitive, emotional, and social factors shaping in-service teachers’ intentions to embrace emerging technologies in education, especially those relating to AI.

## 6. Implication for policy and practice

Our results provide actionable insights for policymakers, educators, and technology developers aiming to facilitate the adoption of ChatGPT among in-service teachers. The strong influence of PEU and PUC highlights the need for user-friendly AI interfaces and practical training programs. We recommend that institutions focus on capacity-building initiatives to enhance teachers’ confidence in using ChatGPT, ensuring its seamless integration into teaching practices. Additionally, YCC significantly impacts adoption, suggesting that collaborative professional development programs and peer-led training workshops can enhance engagement and increase trust in AI tools. The negative impact of TA underscores the importance of providing continuous support systems, including help desks, troubleshooting resources, and professional learning communities, to alleviate fears associated with AI adoption. Although PIU ranked as the least significant factor, ensuring robust data security policies can further strengthen teachers’ confidence in AI-driven tools. We recommend that policymakers develop clear ethical guidelines for AI use in classrooms to address concerns about bias, misinformation, and AI over-reliance. A multi-stakeholder approach, involving education ministries, teacher training institutes, and AI developers, is crucial for building an inclusive, innovative, and AI-ready educational ecosystem that maximizes the benefits of GenAI in teaching and learning.

## 7. Conclusion

Our study provides critical insights into the factors influencing Nigerian in-service teachers’ adoption of ChatGPT, leveraging a hybrid approach that integrates PLS-SEM and ANN. Our results reveal that PEU, PUC, and YCC are the strongest drivers of behavioral intention, emphasizing the role of usability, perceived benefits, and peer influence in AI adoption. Conversely, TA negatively affects adoption, highlighting the need for strategies to reduce fear and uncertainty associated with AI integration. The ANN model captured complex nonlinear interactions, reinforcing the importance of usability as a foundation for perceived usefulness. By combining PLS-SEM’s statistical rigor with ANN’s ability to detect hidden patterns, we offer a comprehensive understanding of AI adoption dynamics. These insights inform policy recommendations, teacher training initiatives, and future research, emphasizing the importance of user-centered AI design and institutional support in fostering sustainable GenAI integration in education. Our findings highlight the need for targeted interventions that address both technical usability and psychological readiness, ensuring that teachers can effectively leverage ChatGPT for instructional purposes.

## 8. Limitations and future directions

Despite our contributions, this study has several limitations that should be addressed in future research. First, our sample was limited to Nigerian in-service teachers, which may restrict generalizability to other educational contexts. Future studies should explore cross-cultural comparisons to examine variations in GenAI adoption. Second, while our hybrid PLS-SEM and ANN approach provided deep insights, it did not capture qualitative perspectives on teachers’ experiences with ChatGPT. We recommend incorporating mixed-method approaches, including focus group interviews, to gain richer contextual insights. Additionally, we focused on behavioral intention (BI) rather than actual usage behavior. Future longitudinal studies tracking teachers’ actual adoption trends over time would enhance our understanding of sustained AI integration. Lastly, further research should explore the role of AI ethics, digital literacy levels, and institutional policies in shaping teachers’ engagement with large language models (LLMs) and GenAI in education. By addressing these gaps, future studies can provide more comprehensive strategies for ensuring AI adoption in diverse educational settings.

## Ethics and consent

Ethical clearance was obtained from the Department of Counseling Psychology and Educational Foundation Ethical Review Board at Tai Solarin University of Education, with approval granted on July 22, 2024, under Ethical Clearance Number ECN 07-2024-15. This approval confirmed our adherence to established ethical standards for studies involving human participants and aligned with the principles outlined in the Declaration of Helsinki, which governs research involving human subjects.

To uphold these ethical standards, we ensured that respondents were fully informed about the purpose of the research, the voluntary nature of their participation, and the confidentiality of their responses. This information was provided alongside the survey, which was shared on professional teachers’ platforms. Respondents could not proceed to complete the online form without first reading and accepting this information. This process guaranteed that written informed consent was obtained electronically before participation, reinforcing their right to decide voluntarily about their involvement. Additionally, we maintained strict confidentiality throughout the study. Responses were securely stored in password-protected digital files and were accessible only to the research team, safeguarding participant privacy. By completing the survey, respondents willingly agreed to participate, fully aware of their rights and the protective measures in place. These ethical commitments underscore our dedication to responsible research practices and the ethical integrity of our study.

## Data Availability

Mendeley Data: Dataset on Large Language Models and GenAI Adoption Among Nigerian Teachers. 10.17632/r444kmwmsy.1 ( Ayanwale, Musa Adekunle et al., 2025). The project contains the following underlying data:
1. ChatGPT Survey-Instrument.docx 2. latent score_for_ANN.xlsx 3.
Main_Dataset_PLS_SEM.xlsx 4. R codes_PLS_SEM_ANN.R Data are available under the terms of the Creative Commons Attribution 4.0 International license (CC-BY 4.0).

## References

[ref63] Aghaziarati A Nejatifar S Abedi A : Artificial Intelligence in Education: Investigating Teacher Attitudes. *AI Tech Behav. Soc. Sci.* 2023;1(1):35–42. 10.61838/kman.aitech.1.1.6

[ref2] Al-kfairy M : Factors impacting the adoption and acceptance of ChatGPT in educational settings: A narrative review of empirical studies. *Appl. Syst. Innov.* 2024;7(6):110. 10.3390/asi7060110

[ref1] Alfadda HA Mahdi HS : Measuring students’ use of zoom application in language course based on the technology acceptance model (TAM). *J. Psycholinguist. Res.* 2021;50(4):883–900. 10.1007/s10936-020-09752-1 33398606 PMC7781650

[ref3] Almogren AS Al-Rahmi WM Dahri NA : Exploring factors influencing the acceptance of ChatGPT in higher education: A smart education perspective. *Heliyon.* 2024;10(11):e31887. 10.1016/j.heliyon.2024.e31887 38845866 PMC11154614

[ref4] Asimov I : *I, robot.* Spectra;2004; vol.1.

[ref5] Athiyaman A : Internet users’ intention to purchase air travel online: an empirical investigation. *Mark. Intell. Plan.* 2002;20(4):234–242. 10.1108/02634500210431630

[ref6] Atlas S : ChatGPT for higher education and professional development: A guide to conversational AI. 2023.

[ref62] Ayanwale MA Adelana OP Bamiro NB : Dataset on Large Language Models and GenAI Adoption Among Nigerian Teachers.[Dataset]. *Mendeley Data.* 2025;V1. 10.17632/r444kmwmsy.1

[ref91] Ayanwale MA Adelana OP Odufuwa TT : Exploring STEAM teachers’ trust in AIbased educational technologies: a structural equation modelling approach. Discover. *Education.* 2024;3(1). 10.1007/s44217-024-00092-z

[ref64] Ayanwale MA Sanusi IT Adelana OP : Teachers’ readiness and intention to teach artificial intelligence in schools. *Comput. Educ.: Artif. Intell.* 2022;3: 100099. 10.1016/j.caeai.2022.100099

[ref65] Baclic O Tunis M Young K : Artificial intelligence in public health: Challenges and opportunities for public health made possible by advances in natural language processing. *Can. Commun. Dis. Rep.* 2020;46(6):161–168. 10.14745/ccdr.v46i06a02 32673380 PMC7343054

[ref7] Bhaskar P Misra P Chopra G : Shall I use ChatGPT? A study on perceived trust and perceived risk towards ChatGPT usage by teachers at higher education institutions. *Int. J. Inf. Learn. Technol.* 2024;41(4):428–447. 10.1108/IJILT-11-2023-0220

[ref8] Bhat MA Tiwari CK Bhaskar P : Examining ChatGPT adoption among educators in higher educational institutions using extended UTAUT model. *J. Inf. Commun. Ethics Soc.* 2024;22(3):331–353. 10.1108/JICES-03-2024-0033

[ref9] Bhullar PS Joshi M Chugh R : ChatGPT in higher education-a synthesis of the literature and a future research agenda. *Educ. Inf. Technol.* 2024;29:21501–21522. 10.1007/s10639-024-12723-x

[ref10] Bhutoria A : Personalized education and artificial intelligence in the United States, China, and India: A systematic review using a human-in-the-loop model. *Comput. Educ.: Artif. Intell.* 2022;3:100068.

[ref66] Biswas SS : Potential Use of Chat GPT in Global Warming. *Ann. Biomed. Eng.* 2023;51:1126–1127. 10.1007/s10439-023-03171-8 36856927

[ref11] Budhathoki T Zirar A Njoya ET : ChatGPT adoption and anxiety: a cross-country analysis utilising the unified theory of acceptance and use of technology (UTAUT). *Stud. High. Educ.* 2024;49:831–846. 10.1080/03075079.2024.2333937

[ref67] Bulathwela S Muse H Yilmaz E : Scalable educational question generation with pre- trainedlanguage models.In International Conference on Artificial Intelligence in Education. Switzerland: Cham: Springer Nature;2023; pp.327–339. Reference Source

[ref12] Cascella M Montomoli J Bellini V : Evaluating the feasibility of ChatGPT in healthcare: an analysis of multiple clinical and research scenarios. *J. Med. Syst.* 2023;47(1):33. 10.1007/s10916-023-01925-4 36869927 PMC9985086

[ref14] Chiang CH Chen WC Kuan CY : Large Language Model as an Assignment Evaluator: Insights, Feedback, and Challenges in a 1000+ Student Course. *arXiv preprint arXiv:2407.05216.* 2024.

[ref13] Chiang CH Lee HY : Can large language models be an alternative to human evaluations?. *arXiv preprint arXiv:2305.01937.* 2023.

[ref15] Choudhury A Shamszare H : Investigating the impact of user trust on the adoption and use of ChatGPT: survey analysis. *J. Med. Internet Res.* 2023;25:e47184. 10.2196/47184 37314848 PMC10337387

[ref16] Cohen J : A power primer. *Psychol. Bull.* 1992;112(1):155–159. 10.1037/0033-2909.112.1.155 19565683

[ref68] Collie RJ Martin AJ : Teachers’ motivation and engagement to harness generative AI for teaching and learning: The role of contextual, occupational, and background factors. *Comput. Educ.: Artif. Intell.* 2024;6: 100224.

[ref17] Davis FD : Perceived usefulness, perceived ease of use, and user acceptance of information technology. *MIS Q.* 1989;13(3):319–340. 10.2307/249008

[ref69] Denny P Khosravi H Hellas A : Can we trust AI-generated educational content? comparative analysis of human and AI-generated learning resources. *arXiv preprint arXiv:2306.10509.* 2023.

[ref70] Dowling M Lucey B : ChatGPT for (finance) research: The Bananarama conjecture. *Fin. Res. Lett.* 2023;53: 103662. 10.1016/j.frl.2023.103662

[ref19] Duong CD Ngo TVN Khuc TA : Unraveling the dark side of ChatGPT: a moderated mediation model of technology anxiety and technostress. *Inf. Technol. People.* 2024. 10.1108/ITP-11-2023-1151

[ref20] Eke DO : ChatGPT and the rise of generative AI: Threat to academic integrity? *J. Responsible Technol.* 2023;13:100060. 10.1016/j.jrt.2023.100060

[ref71] El Naqa I Karolak A Luo Y : Translation of AI into oncology clinical practice. *Oncogene.* 2023;42(42):3089–3097. 10.1038/s41388-023-02826-z 37684407 PMC12516697

[ref72] Essien A Salami A Ajala O : Exploring socio-cultural influences on generative AI engagement in Nigerian higher education: an activity theory analysis. *Smart Learn. Environ.* 2024;11:63. 10.1186/s40561-024-00352-3

[ref21] Fishbein M Ajzen I : Belief, attitude, intention, and behavior: An introduction to theory and research. 1977.

[ref22] Fui-Hoon Nah F Zheng R Cai J : Generative AI and ChatGPT: Applications, challenges, and AI-human collaboration. *J. Inf. Technol. Case Appl. Res.* 2023;25(3):277–304. 10.1080/15228053.2023.2233814

[ref23] Gefen D Karahanna E Straub DW : Trust and TAM in Online Shopping: An Integrated Model. *MIS Q.* 2003;27:51–90. 10.2307/30036519

[ref73] Guo MH Xu TX Liu JJ : Attention mechanisms in computer vision: A survey. *Comput. Vis. Media.* 2022;8(3):331–368. 10.1007/s41095-022-0271-y

[ref25] Hair JF Hult GTM Ringle CM : *A Primer on Partial Least Squares Structural Equation Modeling (PLS-SEM).* 3rd ed. Sage;2022. 10.1007/978-3-030-80519-7

[ref27] Hair JF Risher JJ Sarstedt M : When to use and how to report the results of PLS-SEM. *Eur. Bus. Rev.* 2019;31(1):2–24. 10.1108/EBR-11-2018-0203

[ref24] Hair JF Jr Hult GTM Ringle CM : Partial Least Squares Structural Equation Modeling (PLS-SEM) Using R: A Workbook. 2021.

[ref74] Han J Yoo H Myung J : *Fabric: Automated scoring and feedback generation for essays.* 2023; 10.48550/arXiv.2310.05191

[ref28] Henseler J Ringle CM Sarstedt M : A New Criterion for Assessing Discriminant Validity in Variance-Based Structural Equation Modeling. *J. Acad. Mark. Sci.* 2015;43:115–135. 10.1007/s11747-014-0403-810.2307/30036540

[ref75] Huang H Zheng O Wang D : ChatGPT for shaping the future of dentistry: the potential of multi-modal large language model. Int. J. Oral Sci. 2023;15(1):29. 10.1038/s41368-023-00239-y 37507396 PMC10382494

[ref29] Ivanov S : The dark side of artificial intelligence in higher education. *Serv. Ind. J.* 2023;43(15-16):1055–1082. 10.1080/02642069.2023.2258799

[ref30] Jiang AQ Sablayrolles A Mensch A : Mistral 7B. *arXiv preprint arXiv:2310.06825.* 2023.

[ref31] Jo H Bang Y : Analyzing ChatGPT adoption drivers with the TOEK framework. *Sci. Rep.* 2023;13(1):22606. 10.1038/s41598-023-49710-0 38114544 PMC10730566

[ref76] Jury B Lorusso A Leinonen J : Evaluating llm- generated worked examples in an introductory programming course. Proceedings of the 26th Australasian computing education conference. Association for Computing Machinery, New York, NY, USA(2024), pp.77–86;2024, January. 10.1145/3636243.3636252

[ref77] Kaplan-Rakowski R Grotewold K Hartwick P : Generative AI and teachers’ perspectives on its implementation in education. *J. Interact. Learn. Res.* 2023;34(2):313–338.

[ref32] Kim S Shin J Cho Y : Prometheus: Inducing fine-grained evaluation capability in language models. *The Twelfth International Conference on Learning Representations.* 2023, October.

[ref33] Kim S Suk J Longpre S : Prometheus 2: An open source language model specialized in evaluating other language models. *arXiv preprint arXiv:2405.01535.* 2024.

[ref34] Labadze L Grigolia M Machaidze L : Role of AI chatbots in education: systematic literature review. *Int. J. Educ. Technol. High. Educ.* 2023;20(1):56. 10.1186/s41239-023-00426-1

[ref35] Latif E Zhai X : Fine-tuning ChatGPT for automatic scoring. *Comput. Educ.: Artif. Intell.* 2024;6:100210.

[ref78] Lee D Arnold M Srivastava A : The impact of generative AI on higher education learning and teaching: A study of educators’ perspectives. *Comput. Educ.: Artif. Intell.* 2024;6: 100221.

[ref79] Leiker D Gyllen AR Eldesouky I : Generative AI for learning: Investigating the potential of learning videos with synthetic virtual instructors.In Wang N Rebolledo-Mendez G Dimitrova V editors. Communications in computer and information science: Vol. 1831. Artificial intelligence in education. Posters and late breaking results, workshops and tutorials, industry and innovation tracks, practitioners, doctoral consortium and Blue Sky. Switzerland: Cham: Springer Nature;2023;pp.523–529.

[ref36] Leong L-Y Hew T-S Ooi K-B : An SEM-ANN approach - guidelines in information systems research. *J. Comput. Inf. Syst.* 2024;1–32. 10.1080/08874417.2024.2329128

[ref38] Liébana-Cabanillas F Marinkovi´c V Kalini´c Z : A SEM-neural network approach for predicting antecedents of m-commerce acceptance. *Int. J. Inf. Manag.* 2017;37(2):14–24. 10.1016/j.ijinfomgt.2016.10.008

[ref37] Li J Sun S Yuan W : Generative judge for evaluating alignment. *arXiv preprint arXiv:2310.05470.* 2023.

[ref39] Likert R : A technique for the measurement of attitudes. *Arch. Psychol.* 1932.

[ref80] Lin J Thomas DR Han F : Using large language models to provide explanatory feedback to human tutors. The 24th International Conference on Artificial Intelligence in Education, AIED 2023. *Educational Dialogue Act Classification.* 2023; 10.48550/arXiv.2306.15498

[ref40] Liu Y Iter D Xu Y : G-eval: Nlg evaluation using gpt-4 with better human alignment. *arXiv preprint arXiv:2303.16634.* 2023.

[ref81] Liu Y Iter D Xu Y : G-eval: Nlg evaluation using gpt-4 with better human alignment. *arXiv preprint arXiv:2303.16634.* 2023.

[ref83] Lu H He L Yu H : A Study on Teachers’ Willingness to Use Generative AI Technology and Its Influencing Factors: Based on an Integrated Model. *Sustainability.* 2024;16(16):7216. 10.3390/su16167216

[ref82] Lund BD Wang T Mannuru NR : ChatGPT and a new academic reality: Artificial Intelligence-written research papers and the ethics of the large language models in scholarly publishing. *J. Assoc. Inf. Sci. Technol.* 2023;74(5):570–581. 10.1002/asi.2475

[ref41] Magno F Cassia F Ringle CM : A brief review of partial least squares structural equation modeling (PLS-SEM) use in quality management studies. *TQM J.* 2024;36(5):1242–1251. 10.1108/TQM-06-2022-0197

[ref84] Mesko B Topol EJ : The imperative for regulatory oversight of large language models (or generative AI) in healthcare. *NPJ Digit. Med.* 2023;6(1):120. 10.1038/s41746-023-00873-0 37414860 PMC10326069

[ref42] Misra S Adtani R Singh Y : Exploring the factors affecting behavioral intention to adopt wearable devices. *Clin. Epidemiol. Glob. Health.* 2023;24:101428. 10.1016/j.cegh.2023.101428

[ref85] Nazaretsky T Cukurova M Ariely M : Confirmation bias and trust: human factors that influence teachers’ attitudes towards AI-based educational technology. *In CEUR Workshop Proceedings.* 2021;3042.

[ref43] Nguyen A Ngo HN Hong Y : Ethical principles for artificial intelligence in education. *Educ. Inf. Technol.* 2023a;28(4):4221–4241. 10.1007/s10639-022-11316-w 36254344 PMC9558020

[ref44] Nguyen DM Chiu YTH Le HD : Determinants of continuance intention towards banks’ chatbot services in Vietnam: A necessity for sustainable development. *Sustainability.* 2021;13(14):7625. 10.3390/su13147625

[ref86] Nguyen HA Stec H Hou X Di S, & McLaren BM : Evaluating ChatGPT’s decimal skills and feedback generation in a digital learning game.In Viberg O Jivet I Merino PM Perifanou M, & Papathoma T (Eds.), Lecture notes in computer science: Vol. 14200. Responsive and sustainable educational futures(pp.278–293). Switzerland, Cham: Springer Nature;2023b. 10.1007/978-3-031-42682-7_19

[ref45] Nilsson F Tuvstedt J : GPT-4 as an Automatic Grader: The accuracy of grades set by GPT-4 on introductory programming assignments. 2023.

[ref46] Ooi K-B Tan GW-H : Mobile Technology Acceptance Model: An Investigation Using Mobile Users to Explore Smartphone Credit Card. *Expert Syst. Appl.* 2016;59:33–46. 10.1016/j.eswa.2016.04.015

[ref47] Peng MYP Yan X : Exploring the influence of determinants on behavior intention to use of multiple media kiosks through technology readiness and acceptance model. *Front. Psychol.* 2022;13:852394. 10.3389/fpsyg.2022.852394 35432060 PMC9009310

[ref87] Pozdniakov S Brazil J Abdi S : Large language models meet user interfaces: The case of provisioning feedback. *Comput. Educ.: Artif. Intell.* 2024;7: 100289. 10.1016/j.caeai.2024.100289

[ref48] R Core Team: *R: A language and environment for statistical computing.* Vienna, Austria: R Foundation for Statistical Computing;2021. Reference Source

[ref88] Shahzad MF Xu S Javed I : ChatGPT awareness, acceptance, and adoption in higher education: The role of trust as a cornerstone. *Int. J. Educ. Technol. High. Educ.* 2024;21(1):46. 10.1109/ICASSP39728.2021.9413901

[ref49] Shiau WL Chau PY : Understanding behavioral intention to use a cloud computing classroom: A multiple model comparison approach. *Inf. Manag.* 2016;53(3):355–365. 10.1016/j.im.2015.10.004

[ref50] Taneja A Arora A : Modeling user preferences using neural networks and tensor factorization model. *Int. J. Inf. Manag.* 2019;45:132–148. 10.1016/j.ijinfomgt.2018.10.010

[ref51] Tillmanns T Salomão Filho A Rudra S : Mapping Tomorrow’s Teaching and Learning Spaces: A Systematic Review on GenAI in Higher Education. *Trends High. Educ.* 2025;4(1):2. 10.3390/higheredu4010002

[ref52] Venkatesh V Davis FD : A Theoretical Extension of the Technology Acceptance Model: Four Longitudinal Field Studies. *Manag. Sci.* 2000;46:186–204. 10.1287/mnsc.46.2.186.11926

[ref53] Venkatesh V Morris MG Davis GB : User Acceptance of Information Technology: Toward a Unified View. *MIS Q.* 2003;27:425–478. 10.2307/30036540

[ref94] Wang ES Chou NP : Consumer Characteristics, Social Influence, and System Factors on Online Group-Buying Repurchasing Intention. *J. Electron. Commer. Res.* 2014;15:119.

[ref89] Walter Y : Embracing the future of Artificial Intelligence in the classroom: The relevance of AI literacy, prompt engineering, and critical thinking in modern education. *Int. J. Educ. Technol. High. Educ.* 2024;21(1):15. 10.1186/s41239-024-00448-3

[ref93] Williams JD Henderson M Raux A : The dialog state tracking challenge series. *AI Mag.* 2014;35(4):121–124. 10.1609/aimag.v35i4.2558

[ref54] Wu T Zhang SH : Applications and Implication of Generative AI in Non-STEM Disciplines in Higher Education. *International Conference on AI-generated Content.* Singapore: Springer Nature Singapore;2023, August; pp.341–349.

[ref55] Wu X Duan R Ni J : Unveiling security, privacy, and ethical concerns of ChatGPT. *Journal of Information and Intelligence.* 2024;2(2):102–115. 10.1016/j.jiixd.2023.10.007

[ref56] Yang Y Wang X : Modeling the intention to use machine translation for student translators: An extension of Technology Acceptance Model. *Comput. Educ.* 2019;133:116–126. 10.1016/j.compedu.2019.01.015

[ref57] Yuan P Feng S Li Y : BatchEval: Towards Human-like Text Evaluation. *arXiv preprint arXiv:2401.00437.* 2023.

[ref92] Zhai X Krajcik J : Artificial Intelligence-Based STEM. *Uses of Artificial Intelligence in STEM Education.* 2024;1. 10.1093/oso/9780198882077.003.0001

[ref58] Zhang X Zhang X Liu H : Reflections on Enhancing Higher Education Classroom Effectiveness Through the Introduction of Large Language Models. *J Mod Educ Res.* 2024;3. 10.53964/jmer.2024019

[ref59] Zheng L Chiang WL Sheng Y : Judging llm-as-a-judge with mt-bench and chatbot arena. *Adv. Neural Inf. Proces. Syst.* 2023;36:46595–46623.

[ref90] Zhou C Li Q Li C : *A comprehensive survey on pretrained foundation models: a history from BERT to ChatGPT.* Int: J. Mach. Learn. & Cyber;2024. 10.1007/s13042-024-02443-6

[ref60] Zogheib S Zogheib B : Understanding University Students’ Adoption of ChatGPT: Insights from TAM, SDT, and Beyond. *J. Inf. Technol. Educ.: Res.* 2024;23:025.

[ref61] Zou M Huang L : To use or not to use? Understanding doctoral students’ acceptance of ChatGPT in writing through technology acceptance model. *Front. Psychol.* 2023;14:1259531. 10.3389/fpsyg.2023.1259531 37954179 PMC10637381

