# Supplementary material for: Large language models and GenAI in education: Insights from Nigerian in-service teachers through a hybrid ANN-PLS-SEM approach
Source: F1000Res. 2025 Mar 4;14:258. [Version 1] doi: 10.12688/f1000research.161637.1 (PMC12359025; doi:10.12688/f1000research.161637.1)
Supplement: Peer review history

# Reviewer report: Reviewer response for version 1

**DOI:** 10.5256/f1000research.177692.r376461

**Published:** 2025-04-30

**Authors:** Sarin Sok

**Affiliations:** 1 University of Puthisastra,, Phnom Penh, Cambodia

Dear Authors, 

 I highly appreciate your great work, which is of my great interest.

 Drawing on a hybrid analytical approach (Partial Least Squares Structural Equation Modeling and Artificial Neural Networks), this study appeared to offer significant insights into Nigerian pre-service teachers’ behavioural intention to use GenAI in education drawing upon the TAM framework, and may also contribute to burgeoning prior literature. It also highlighted key recommendations for both policy and practice which are of paramount importance for such an emerging topic–GenAI in pre-service teacher education. However, there is a need to address some identified weaknesses to improve the overall quality of the article. 

 - While this study comprehensively discussed the theoretical framework (TAM) used to investigate pre-service teachers’ behavioural intention to utilize GenAI in education, and offered a nuanced discussion over the hypothesis development used in this study, and relevant studies in the global context, there is a lack of contextual review. Therefore, it is of benefit to review relevant prior studies discussing the use of GenAI in Nigerian education, especially in the context of pre-service education. This may also provide better context for the “discussion section” by comparing the current study with existing ones in Nigeria, or, at least, in the developing contexts. 

 - While the current study highlighted that experts in AI in the education field were invited to review, and validate the questionnaire, it left me wondering how many experts? Did the pilot study be conducted to ensure its validity and reliability beyond the current validation method (Table 2)? Moreover, it would be of benefit to specify the exact months and dates during which the questionnaire was distributed, rather than only commenting that it was for two months. 

 - The last three sentences (this approach….was accepted) in the method of data collection part seems to repeat the “study instrument” (The survey remained…..to contribute). Therefore, it would be great to revise these two sections. 

 - The method of self-reported data via Google-Forms survey used in this study may contribute to bias responses, and that it is important for the authors to acknowledge this limitation by suggesting future research to address. 

 - While this article is generally well-written and academically sound, the use of acronyms such as AI, GenAI, LLMs …… was not consistent across the manuscript. Academically, the full term for an acronym should be spelled out upon its first use, the acronym can be then used consistently.

 I hope you find my comments helpful. 

 Thank you!

Is the work clearly and accurately presented and does it cite the current literature?

Yes

If applicable, is the statistical analysis and its interpretation appropriate?

I cannot comment. A qualified statistician is required.

Are all the source data underlying the results available to ensure full reproducibility?

Yes

Is the study design appropriate and is the work technically sound?

Yes

Are the conclusions drawn adequately supported by the results?

Yes

Are sufficient details of methods and analysis provided to allow replication by others?

Yes

Reviewer Expertise:

Educational technology, higher education, GenAI in education and research, teacher education

I confirm that I have read this submission and believe that I have an appropriate level of expertise to confirm that it is of an acceptable scientific standard.

# Reviewer report: Reviewer response for version 1

**DOI:** 10.5256/f1000research.177692.r373182

**Published:** 2025-04-04

**Authors:** Damola Olugbade

**Affiliations:** 1 First Technical University, Ibadan, Nigeria

The study presents a structured and relevant exploration of the topic titled Large Language models and GenAI in education: Insights from Nigeria in-service teachers through a hybrid ANN-PLS-SEM approach.

 This research will contribute valuable insights to the computer education. Its objectives are articulated, and the findings are well presented, aligning with broader discussions on digital education and technology integration.

 The methodology is appropriately selected, with a structured approach that enhances the study’s reliability. Additionally, the discussion effectively connects the results to existing theoretical and practical implications, ensuring clarity in interpretation.

 However, there are areas where the study could be strengthened. The methodology section would benefit from greater transparency, particularly in **detailing** the research design, sample selection, and data collection procedures.

 Additionally, the discussion of findings could be expanded to highlight their practical implications, particularly **with educational policy and practice**. A more comparative perspective, positioning the findings within the broader research landscape, could also enrich the discussion.

 While the overall quality of the study is commendable, a more focused review of specific sections could provide deeper insights if needed.

Is the work clearly and accurately presented and does it cite the current literature?

Yes

If applicable, is the statistical analysis and its interpretation appropriate?

Yes

Are all the source data underlying the results available to ensure full reproducibility?

Yes

Is the study design appropriate and is the work technically sound?

Yes

Are the conclusions drawn adequately supported by the results?

Yes

Are sufficient details of methods and analysis provided to allow replication by others?

Yes

Reviewer Expertise:

Educational Technology, Technology Enhanced Learning, Computer Education, AI in Education

I confirm that I have read this submission and believe that I have an appropriate level of expertise to confirm that it is of an acceptable scientific standard.

## References

[1] : Large language models and GenAI in education: Insights from Nigerian in-service teachers through a hybrid ANN-PLS-SEM approach. *F1000Research* .2025;14: 10.12688/f1000research.161637.1 10.12688/f1000research.161637.1 40831547 PMC12359025
